# A 360 degree mixed-methods evaluation of a specialized COVID-19 outpatient clinic and remote patient monitoring program

**DOI:** 10.1186/s12875-022-01734-7

**Published:** 2022-06-13

**Authors:** Stacie Vilendrer, Anna Lestoquoy, Maja Artandi, Linda Barman, Kendell Cannon, Donn W. Garvert, Douglas Halket, Laura M. Holdsworth, Sara Singer, Laura Vaughan, Marcy Winget

**Affiliations:** Department of Medicine, Stanford University School of Medicine, 1265 Welch Rd, Stanford, CA 94305 USA

**Keywords:** COVID-19, Outpatients, Remote patient monitoring, Retrospective studies, Health services, Telemedicine, Academic medical centers

## Abstract

**Background:**

Our goals are to quantify the impact on acute care utilization of a specialized COVID-19 clinic with an integrated remote patient monitoring program in an academic medical center and further examine these data with stakeholder perceptions of clinic effectiveness and acceptability.

**Methods:**

A retrospective cohort was drawn from enrolled and unenrolled ambulatory patients who tested positive in May through September 2020 matched on age, presence of comorbidities and other factors. Qualitative semi-structured interviews with patients, frontline clinician, and administrators were analyzed in an inductive-deductive approach to identify key themes.

**Results:**

Enrolled patients were more likely to be hospitalized than unenrolled patients (*N* = 11/137 in enrolled vs 2/126 unenrolled, *p* = .02), reflecting a higher admittance rate following emergency department (ED) events among the enrolled vs unenrolled, though this was not a significant difference (46% vs 25%, respectively, *p* = .32). Thirty-eight qualitative interviews conducted June to October 2020 revealed broad stakeholder belief in the clinic’s support of appropriate care escalation. Contrary to beliefs the clinic reduced inappropriate care utilization, no difference was seen between enrolled and unenrolled patients who presented to the ED and were not admitted (*N* = 10/137 in enrolled vs 8/126 unenrolled, *p* = .76). Administrators and providers described the clinic’s integral role in allowing health services to resume in other areas of the health system following an initial lockdown.

**Conclusions:**

Acute care utilization and multi-stakeholder interviews suggest heightened outpatient observation through a specialized COVID-19 clinic and remote patient monitoring program may have contributed to an increase in appropriate acute care utilization. The clinic’s role securing safe reopening of health services systemwide was endorsed as a primary, if unmeasured, benefit.

**Supplementary Information:**

The online version contains supplementary material available at 10.1186/s12875-022-01734-7.

## Background

The COVID-19 pandemic dramatically increased the demand for telemedicine and telehealth services [1–3], including in outpatient settings to support patients with confirmed or suspected COVID-19 [4]. Several health systems have introduced remote patient monitoring (RPM) programs as a vital adjunct to in-person COVID-19 clinical care. Such programs seek to guide patients to the appropriate level of care, whilst minimizing unnecessary pathogen exposure to clinical staff and non-COVID-19 patients [5–12].

RPM directs patient data collected in non-clinical settings to clinical teams providing care oversight [13, 14]. It has traditionally been used to guide care for patients with chronic disease [14–21], and its impact may depend on the disease and monitoring approach [15, 22]. Outside of chronic disease, the use of RPM to manage short-term conditions has been primarily limited to post-operative care [14, 23]. Prior to COVID-19, its use in acute infectious illness was virtually non-existent.

COVID-19 expanded RPM possibilities from both the clinical [5–9, 24] and technical [25, 26] perspectives. Recent RPM efforts have combined digital and/or sensor technology (i.e., pulse oximetry) with clinical oversight to determine the need for care escalation. Evaluations of these efforts thus far are limited. Most notably, RPM enrollment reduced 30 day hospital readmissions in COVID-19 patients discharged from the hospital [11, 12]; similar findings were also noted for cancer patients with COVID-19 [27]. Another evaluation found an association between RPM enrollment and a reduced admittance rate following an ED visit [5]. Patient engagement and satisfaction with programs have also been documented [5–7].

This nascent body of work suggests that RPM programs are acceptable to patients and may decrease undesirable healthcare utilization, though additional evaluation is needed to understand how perceived benefits amongst stakeholders compare with actual care utilization and patient outcomes. Such analysis can inform the future direction of such programs, particularly given the non-trivial resources they require. We evaluated a specialized COVID-19 clinic with an integrated RPM program in an academic medical center using a mixed-methods approach. This approach included quantifying the program’s impact on acute care utilization and patient outcomes as well as qualitatively analyzing patient, provider, and administrator perspectives of the program.

## Methods

### Design

We conducted a convergent mixed methods [28] evaluation of a dedicated COVID-19 outpatient clinic at a large academic medical center, Stanford Health Care (Palo Alto, CA, USA). A quantitative retrospective cohort analysis matched patients enrolled and unenrolled in the clinic to understand the clinic’s impact on downstream emergency department (ED) utilization and hospitalization rates. Semi-structured interviews with patients, frontline clinicians, and administrative stakeholders and subsequent analysis were guided by Proctor et al. (2011) Outcomes for Implementation Research [29]. The Stanford Institutional Review Board approved the present retrospective evaluation and determined it did not meet the definition of human subjects research (Protocol #56054). Informed consent was obtained from all interview participants.

### Specialized COVID-19 “CROWN” clinic with RPM

In April 2020, Stanford Health Care (SHC) launched a specialized COVID-19 clinic called CROWN (standing for Care and Respiratory Observation of patients With Novel coronavirus) to provide RPM to adult outpatients recently diagnosed with COVID-19 [30]; this clinic combined with RPM services entail the intervention that is the focus of this evaluation. Any positive COVID-19 laboratory test throughout the health system went into a central pool monitored by ED nurses. Patients with a positive COVID-19 test and fewer than 14 days of symptoms were offered enrollment in the program that included periodic check-ins by phone and/or video. The frequency and method of communication (phone or video) was based on a risk stratification tool developed by lead clinicians, which incorporated age, pre-existing conditions, clinical severity of illness and the clinical course of disease to place patients into low, medium and high risk categories (Additional file 1: Appendix A1). These tiers were used to guide the frequency of outreach through phone calls and video visits as well as the distribution of pulse oximeters to medium and high-risk patients (Additional file 1: Appendix A2). Clinicians at any point could designate a patient receive a higher level of care (e.g. shorter intervals between outreach), including escalation to in-person care at CROWN or the ED if needed. CROWN clinicians followed patients from the date of their enrollment to 14–21 days from first symptoms and provided patients a dedicated phone number to contact the clinic if concerns or questions arose. Biometric data was shared verbally by the patient in the course of each encounter and was not electronically transmitted. Further details regarding the risk stratification tool and RPM outreach protocol have been previously described [30].

### Quantitative evaluation - creation of the matched retrospective cohort

We extracted electronic health record demographic and acute care utilization data of all patients who received a positive test result from a Stanford testing facility between May 1, 2020 and September 30, 2020. The time between the COVID-19 test result date and enrollment varied, with a median of 2 days. To ensure a similar duration of disease progression for the purposes of matching, a “pseudo enrollment” date defined as 2 days following a COVID-19 test result was assigned to the unenrolled comparator group. Patients were excluded if they were under age 18, lived more than 50 miles from Stanford Hospital or had a missing zip code, or if their positive test date was after September 2, 2020, to ensure complete follow-up of all patients; follow-up was defined as 28 days following the date of program enrollment (or pseudo-enrollment). Patients were also excluded if they were hospitalized in the 7 days preceding and inclusive of the enrollment (or pseudo-enrollment) date, a decision made to focus the evaluation on the intervention’s effectiveness at directing patients to the appropriate level of care early in their disease course.

Manual chart reviews were conducted by physician authors (with regular audits by author SV). These were used to identify the binary presence of comorbidities based on the COVID-19 risk stratification protocol (Additional file 1: Appendix A) in addition to emergency room and hospitalization encounters in the exclusion and observation periods that were not captured in the extracted dataset but were viewable within the electronic health record (Epic, Wisconsin, USA).

The matched cohort was finalized by matching each eligible enrolled patient to an unenrolled patient considering home distance from hospital (0–15, > 15–30, > 30–50 miles), health system affiliation (Stanford academic, Stanford non-academic made up of affiliated community practices, and unaffiliated), insurance type (private, Medicare, other) and race/ethnicity (Hispanic, White, Asian, other), age (closest match), and binary presence of relevant comorbidities (technical details in Additional file 1: Appendix B).

### Quantitative data analysis

The outcomes of interest for the matched analysis were COVID-related unique patient and total ED encounters, unique patient and total hospital admissions (inclusive of an admission to observation status), and death in the 28 days following the date of program enrollment (or pseudo-enrollment). The ratio of a positive admission following an ED visit was reported for each group (defined as # inpatient admissions / (# inpatient admissions + # ED encounters)); these rates were statistically compared using a mixed effects logistic regression model with a random effect for patient. We also compared rates of COVID-related ED and hospital admissions by risk severity level for enrolled patients as an informal validation of the clinical risk assignment protocols. Analyses were conducted using R version 4.0.5 software and SAS version 9.4 software. Statistical significance was set at 0.05; *p*-values were adjusted for multiple comparisons using Tukey-Kramer correction where appropriate. Poisson regressions were used to determine statistical significance for counted events, including to validate the risk stratification tool where risk level was an explanatory variable and COVID-related ED and hospital admissions were outcomes.

We conducted a detailed chart review to extract clinical details of all COVID-19-related ED encounters and hospitalizations to provide a descriptive analysis only, as our evaluation was not powered to detect changed at this level. Data extracted included chief complaint, referring party (self, clinic, other), and suboptimal oxygenation (defined as a pulse oximeter reading of 95% or below at first clinical presentation) and are described as a percentage of a given event type. Given ever-shifting COVID-19 treatment guidelines at the time, for the purposes of analysis and discussion, we relied on a crude definition of “appropriate care” in which a hospital admission followed an ED encounter.

### Qualitative stakeholder groups & data collection

We interviewed clinic stakeholders including enrolled patients, providers, and administrators. Interviews with unenrolled patients were not pursued given their experiences have been described elsewhere [31, 32], as well as our need to direct limited resources to better understand the clinic’s implications for the purposes of quality improvement. Enrolled patients were selected to include a diverse representation of gender, primary language spoken, and COVID-19 risk severity level from a list of those who completed clinic enrollment within the previous 3 weeks with no prior hospitalization for COVID-19. Patients were approached by telephone, given a verbal description of the evaluation, and gave their informed consent to participate in the interview. All providers who made up the core clinical team were contacted for interview, as were key administrators who were familiar with ambulatory clinical operations, acute care clinical operations, and finances related to clinic operations.

Semi-structured interview protocols were adapted for each stakeholder group based on its relevant perspective (Additional file 1: Appendix C). Interviews were conducted by either of two researchers (AL, SV), recorded and transcribed for analysis. Transcripts of interviews conducted in Spanish were first transcribed in Spanish and then translated to English by health system affiliates with professional translation training.

### Qualitative analysis

We conducted thematic analysis of interviews using a combined inductive and deductive approach with separate codebooks developed for each stakeholder group. The deductive codes were derived from the topic guide content and key implementation outcomes [29]. All transcripts were imported into NVivo (released March 2020) for analysis. Two coders (SV, ASL) first independently coded one transcript then met to discuss and align coding practice. The process was repeated until the researchers agreed coding alignment was achieved for each stakeholder group. Coders continued to independently code transcripts and met regularly to review emerging themes and revise the codebook [33]. Coded data were then summarized into a thematic matrix with color-coding to identify positive and negative sentiments where rows represented individual participants and columns represented key themes; this visualization further supported the identification of intra and inter- group convergence and divergence of opinion [34].

## Results

### Quantitative analysis

#### Characteristics of enrolled and unenrolled patients

A total of 719 patients (89 Stanford academic, 20 Stanford non-academic, and 610 unaffiliated) enrolled in the clinic during the inclusion period. After applying exclusion and selection criteria for all populations, 137 eligible enrolled patients and 126 unenrolled matched patients were included in the analysis. There was no statistically significant difference between enrolled and unenrolled patients in terms of key demographic characteristics, suggesting successful matching of the groups on these variables (Table 1**)**. There was also no statistically significant difference in enrolled and unenrolled patients who were hospitalized for non-COVID-19 related reasons, suggesting that these groups were reasonably balanced regarding comorbidities and their general hospital utilization (Additional file 1: Appendix D).

**Table 1:** Characteristics of Enrolled and Unenrolled Patients in the Specialized COVID-19 Clinic

Characteristic	Enrolled *n* = 137 (%)	Unenrolled *n* = 126 (%)	***p***-value
**Age**^**a**^			*p* = .79
18–49	43 (31.4)	43 (34.1)	
50–59	45 (32.8)	39 (31.0)	
60–69	35 (25.5)	35 (27.8)	
70+	14 (10.2)	9 (7.1)	
**Gender**			*p* = .62
Female	76 (55.5)	66 (52.4)	
Male	61 (44.5)	60 (47.6)	
**Race/Ethnicity**			*p* = .98
Hispanic	65 (47.4)	62 (49.2)	
White	28 (20.4)	23 (18.3)	
Asian	11 (8.0)	9 (7.1)	
Black or African American	3 (2.2)	2 (1.6)	
Other	30 (21.9)	30 (23.8)	
**Insurance**			*p* = .72
Private	70 (51.1)	66 (52.4)	
Uninsured/Medicaid/Other	46 (33.6)	45 (35.7)	
MEDICARE	21 (15.3)	15 (11.9)	
**1 or more comorbidities**^**b**^			*p* = .54
Present	55 (40.1)	46 (36.5)	
Not Present	82 (59.9)	80 (63.5)	
**Health system affiliation**			*p* = .88
Stanford academic	53 (38.7)	45 (35.7)	
Stanford non-academic	17 (12.4)	17 (13.5)	
Unaffiliated	67 (48.9)	64 (50.8)	
**Distance from Stanford Hospital**			*p* = .91
0–15 miles	99 (72.3)	93 (73.8)	
> 15–30 miles	30 (21.9)	25 (19.8)	
> 30–50 miles	8 (5.8)	8 (6.3)	

^a^Age at the time of clinic enrollment or pseudo-enrollment

^b^Binary presence of comorbidities followed clinic-developed protocols and included immunocompromised status, moderate or severe asthma, chronic lung disease, cirrhosis, diabetes, severe obesity (BMI > 40), cardiovascular disease including hypertension, chronic kidney disease, or pregnancy

#### Covid-19 related ED encounters and hospitalizations

The enrolled and unenrolled groups had similar proportions of unique patients with a COVID-related ED-only encounter (7% enrolled vs 6% unenrolled; *p* = .76) as well as the total number of these encounters (13 enrolled and 9 unenrolled, *p* = .51) (Table 2). A larger proportion of enrolled patients had COVID-related hospitalizations, however, than unenrolled (8% vs 2%; *p* = .02); the enrolled patients also had a higher number of total hospitalizations (11 vs 3, *p* = .04). Chart review showed each admission event was preceded by an ED event (and direct admissions by primary care providers are not supported in this system), this reflects an increased admittance rate of 46% in the enrolled group (11 admittances of 24 possible events) versus 25% in the unenrolled group (3 admittances of 12 possible events). This could represent more appropriate use of the ED by enrolled patients, though this difference was not statistically significant (*p* = .32). Finally, there was no difference in length of stay during the observed hospitalizations (*p* = .67) or in mortality (*p* > .99).

**Table 2:** COVID-Related Events Within a 28-Day Observation Period by Enrollment Status in the Specialized COVID-19 Clinic

Events During Observation Period	Enrolled*n* = 137 (%)	Unenrolled*n* = 126 (%)	***p***-value
**Patients with ED-only events**^**a**^	10 (7.3)	8 (6.3)	*p* = .76
**Total ED-only events**	13	9	*p* = .51
**Patients with hospital events**	11 (8.0)	2 (1.6)	*p* = .02
**Total hospital events**	11	3	*p* = .04
**Deceased from any cause**^**b**^	1 (0.7)	1 (0.8)	*p* > .99
	**Ave. days (std dev), min-max**	**Ave. days (std dev), min-max**	
**Length of stay for hospital events**	7.3 (7.7) 2–28	4.0 (1.7) 3–6	*p* = .67

^a^Emergency department (ED)-only events include patients who were seen in the ED and subsequently discharged without admission to inpatient or observation status

^b^Chart reviews suggest all deaths were related to COVID-19

Chart reviews revealed a total of 6 ED encounters for asymptomatic repeat COVID-19 testing, (4 of 13; 31% ED visits in the enrolled and 2 of 9; 22% in the unenrolled). Events in which patients self-referred to acute care and were not admitted were similar between enrolled and unenrolled patients (8 of 13; 62% vs 6 of 9; 67%, respectively). However, events in which patients self-referred to acute care and were ultimately admitted were slightly higher in the enrolled than in the unenrolled group (11 of 24; 46% vs 3 of 12; 33%, respectively). Finally, all admission events for unenrolled patients were associated with poor oxygen status (defined as a pulse oximeter reading of 95% or below) at first clinical presentation (3 of 3; 100%), whereas this proportion was slightly lower for enrolled patients (9 of 11; 82%).

#### Validation of COVID-19 risk severity clinic protocol

Most enrolled patients were determined to have low (39%) or medium (43%) COVID-19 Risk Severity based on the clinic protocols (Table 3). There was not a statistically significant difference between risk severity levels and COVID-19-related ED visits or admissions after adjusting for multiple comparisons. However, there was a statistically significant difference between high and low risk severities in rate of ED-only events (*p* = .05) and combined ED and hospital admission events (*p* < .01), thus suggesting the risk stratification tool (Additional file 1: Appendix A) holds some validity.

**Table 3:** Enrolled Patients COVID-related Events Within the 28-Day Observation Period by COVID-19 Risk Severity

Type of COVID-related Event	Enrolled Patients (***n*** = 137)			***p***-value
	Low COVID-19 Risk Severity ***n*** = 53 (%)	Medium COVID-19 Risk Severity ***n*** = 59 (%)	High COVID-19 Risk Severity ***n*** = 25 (%)	
**Patients without any event**	49 (92.5)	51 (86.4)	18 (72.0)	
**Patients with ED-only events**	1 (1.9)	6 (10.2)	3 (12.0)	*p* = .11
**Patients with hospital events**	3 (5.7)	3 (5.1)	5 (20.0)	*p* = .08
**Patients with any ED-only and/or hospital events**	4 (7.5)	8 (13.6)	7 (28.0)	*p* = .06
	**# Encounters**	**# Encounters**	**# Encounters**	
**Total ED-only events**	1	6	6	
**Total hospital events**	3	3	5	
**Combined total ED-only or hospital events**	4	9	11	
	**rate (std dev.)**	**rate (std dev.)**	**rate (std dev.)**	
**Rate of ED-only events**	0.02 (0.14)	0.10 (0.30)	0.24 (0.83)	*p* = .05^a^
**Rate of hospital events**	0.06 (0.23)	0.05 (0.22)	0.20 (0.41)	*p* = .10
**Rate of combined ED-only or hospital events**	0.08 (0.26)	0.15 (0.41)	0.44 (0.92)	*p* < .01^b^

^a^Adjusting for multiple comparisons (Tukey-Kramer), the high severity group had nearly a significant difference compared to the low severity group, *p* = .052

^b^Adjusting for multiple comparisons (Tukey-Kramer), the high severity group had a significantly higher rate than the low severity group (*p* < .01) and had nearly a significant difference compared to the medium severity group, *p* = .052

### Interview findings

#### Characteristics of interviewed stakeholder groups

We conducted a total of 38 qualitative interviews June to October 2020 across the three stakeholder groups, including 21 patients, 9 (of 12 available) providers, and 8 (of 12 available) administrative stakeholders, 2 of whom had overlapping provider and administrative responsibilities and were therefore interviewed with both the provider and administrator protocols. Patient characteristics were predominantly female (67%), Hispanic/Latino (52%), Spanish speaking (52%) and were moderate risk based on clinical criteria (48%), though low and high risk patients were also represented (33, 19%, respectively). Reflecting the personnel serving in the CROWN clinic, providers were predominantly female (78%), physicians (56%) while administrators were also predominantly female (75%) and had diverse areas of operational focus (Table 4). Duration of interviews varied low to high (median) across stakeholder groups: 10–27 (19), 18–45 (25), 17–39 (24) minutes for patients, providers and administrators, respectively. The data below focuses on key themes related to care utilization that complement the quantitative analysis above, system considerations impacting the perceived benefits of the clinic, and clinic features impacting the acceptability of the clinic from patient and provider perspectives. We compare findings across methods to gain insight where applicable.

**Table 4:** Patient, Provider, and Administrative Stakeholder Characteristics

Characteristics	Patients ***n*** = 21 (%)	Providers ***n*** = 9 (%)	Administrators ***n*** = 8 (%)
**Gender**			
*Male*	7 (33)	2 (22)	2 (25)
*Female*	14 (67)	7 (78)	6 (75)
**Race/Ethnicity**			
*Hispanic/Latino*	11 (52)		
*Black Non-Hispanic*	1 (5)		
*White Non-Hispanic*	6 (29)		
*Unknown*	3 (14)		
**Primary Language Spoken**			
*English*	10 (48)		
*Spanish*	11 (52)		
**Risk Severity Level**			
*Low*	4 (19)		
*Moderate*	10 (48)		
*High*	7 (33)		
**Provider Type**			
*Physician*		5 (56)	
*Physician’s Assistant*		3 (33)	
*Nurse Practitioner*		1 (11)	
**Operational Focus**			
*Finance*			2 (25)
*Clinic Operations*			3 (38)
*Executive*			2 (25)
*Acute Care Liaisons*			1 (13)

#### Perceptions and utilization patterns suggest an increase in appropriate care escalation

Both providers and administrators believed the clinic played a crucial role in identifying patients who required care escalation but who may not have otherwise sought or been able to access this care. Providers felt this to be particularly important given the nature of the COVID-19 illness where low blood oxygen levels can be present without associated symptoms, so called “silent hypoxia”. Such cases were detected through symptom monitoring and pulse oximeter information, which was “… super helpful. We do a resting and an ambulatory pulse ox. And then if the O2 sat is worrisome, we either have the patient come to clinic or send them to the emergency room” (Provider 3).

Patients who had access to pulse oximetry overwhelmingly reported the devices were easy to operate, and they used them approximately 1–3 times a day. One patient explicitly described using the device to determine whether to seek care (though no patient interviewed reported the need for escalation of care). Patients expressed universally positive sentiment in having the pulse oximeter as a resource during their illness.

Another mechanism that may have increased appropriate care utilization was the interim step that an in-person evaluation in CROWN offered in place of the ED:

*…I've said, ‘You need to go to the ER,’ and [patients] are like, ‘No, I'm not going to the ER.’ Then I say, ‘Well, if you refuse to go there, I can offer you the CROWN in person, but I might still tell you to go to the ER [emergency department] after I see you.’ They seem more willing to do that than to go to the ER.’ (Provider 2)*

Some patients expressed concerns about possibly being contagious and appreciated the remote care and streamlined in-person care the clinic offered.

These perceptions were supported by the quantitative data described above in which enrolled patients had a significantly higher number of inpatient admissions as well as a higher admittance rate upon presenting to the ED. The lack of a significant difference in length of stay between enrolled and unenrolled patients does not support the notion that patients presented for care earlier because of the program. Clinic providers were a source of referral to ED evaluation in one third (8 of 24 referrals) of the encounters for enrolled patients, suggesting the clinic played a substantial role in escalation of care.

#### Perceptions of a reduction in inappropriate care escalation without corresponding quantitative evidence

Providers and administrators believed the clinic also played a strong role in redirecting patients away from inappropriate care, specifically in the ED. One administrator shared he would “panic” without this resource given it was the primary place to send patients with confirmed or suspected COVID-19 (Administrator 1). Another provider reported that keeping patients out of the ED had become an unexpected predominant role of the clinic:

*We thought that we would be picking up hypoxia that people weren’t noticing and sending them to the ER. And instead, we’re almost doing the opposite where we have patients who may feel a little shortness of breath and then they get their [oxygen saturation] and it’s 99% [optimal] and that actually helps me keep people at home. (Provider 5)*

Without the clinic, providers perceived that patients would be otherwise “completely lost” given restricted access for potentially contagious persons and the reported reluctance of PCPs and specialists to see these patients in person (Provider 2). Several patients corroborated these challenges in accessing primary care, stating their doctors were either slow to return their calls and/or were unable to physically see them in regular clinic: “…they [clinic] said no, that they could not attend to me unless it was an emergency” (Patient 10). CROWN was perceived by providers as offering “everything the patient needs in one place,” including imaging, labs, and other studies, thereby optimizing safety and efficiency (Provider 4).

Despite these beliefs and reported behaviors, the actual utilization data described above does not corroborate these beliefs, as there was no difference between enrolled and unenrolled ED-only utilization. Indeed, chart reviews suggested similar numbers between enrolled and unenrolled patients for an inappropriate ED presentation –asymptomatic repeat COVID-19 testing—despite provider efforts to educate patients during their enrollment.

#### Systems considerations impacting perceived value of the clinic

Several administrators and providers reported the clinic’s greatest impact was possibly its role in supporting access to health services for non-COVID patients. They reported that safety was increased throughout the system by directing potentially infected patients to a single site for non-acute services, thereby facilitating a gradual reopening of services following the initial lockdown:

*Our ability to reopen all the sites…was dependent on the fact that we had a place that we could also appropriately case manage and track people with COVID …So the financial viability of the clinic itself can't be measured in the clinic financial performance, but the rest of organization’s capability to keep pace or open up quickly and stay open. (Administrator 7)*

Administrators also reported challenges capturing reimbursement for the clinic due to structural challenges. Having been rapidly launched, the clinic lacked its own budget and instead operated under the umbrella of its neighboring urgent care clinic with “borrowed” resources (Admin 3). Many RPM activities, such as outreach by non-provider staff were not reimbursable, and when they were, the patient mix reportedly skewed towards the uninsured or underinsured, further limiting reimbursement.

#### Clinic features impacting acceptability of the specialized COVID-19 clinic

Patients and providers described how several clinic characteristics impacted their perceived acceptability of the clinic (Table 5). Patients and providers both expressed generally favorable perceptions related to improved access to care, benefits of patient education, benefits of concentrating COVID-19 expertise within a specialized clinic, support for mental health services, and use of pulse oximeters.

**Table 5:** Clinic Features Impacting Acceptability of the Specialized COVID-19 Clinic Across Patients and Providers

Clinic Feature – Definition & Summary of Findings	Illustrative Patient Quotation(s)	Illustrative Provider Quotation(s)
**Access to care** – *The ease with which patients access services through CROWN* Largely favorable perceptions across patients and providers, particularly given barriers in accessing traditional primary care	“I prefer the CROWN Clinic…because I have to wait a very long time…to talk with my primary care physician. Sometimes they are too busy to answer the phone or especially in, in the time of COVID, they don’t have enough, people to answer the phone…they [CROWN clinic] answered my call right away.” -Patient 9	“…Frankly, just having any single phone number that they know they can reach someone without going to the emergency room, [is] probably the single most important thing. Easy access, well-structured access, as you probably know in healthcare in general, that’s one of the biggest problems, just the friction around access, interacting with a care team when you have a problem.” -Provider 5
**Frequency of outreach** – *The cadence of telephone and video encounters based on the clinic’s risk stratification protocol* Largely favorable perceptions in patients and mixed perceptions in physicians who questioned whether proactive outreach was the best use of scarce resources	“Interviewer: How did you feel about the frequency of these phone calls? Patient: Very good, because they were following up on my health.” -Patient 2	“I think the risk stratification tool has worked very well for us to determine how often patients need to be followed.”-Provider 3 “I talked to patients along the [entire] risk strata…it seemed to be overkill…if we had [un]limited resources, great, but we have tons of…non-COVID patients that have poor access [to care]…[this is] not the right level for the need, but I think the general idea of a care pathway is a good thing.”-Provider 6
**Patient education** – *Role of CROWN clinicians in providing education regarding the disease* Largely favorable perceptions across patients and providers who felt publicly available information and county outreach fell short of COVID-19 patient needs	“…I could speak with them…I was also able to have my uncertainties addressed and everything and so I could ask questions… and it was good…” –Patient 10	“And then, patients have many questions, for example, about when they can return to work, what to do with their family’s safety, how to access resources that they might need for food or for places for their family to stay and social work type needs. And those questions take a ton of time to answer.” -Provider 7
**Specialist COVID-19 team –** *CROWN clinician expertise in providing COVID-19 care* Largely favorable perceptions across patients who felt their questions were answered relative to other health settings and providers who describe significant time involved in developing and maintaining COVID-19 expertise	“…the doctors, nurses and everybody…they understand everything…I’m still going there …because I’m a research patient too…but I had a very good experience. Everybody is nice. They are taking care of me. They are very responsible. They answered all my questions, so I’m very happy, and I’m very glad with the research [participation].” -Patient 9	“I think you do want to have a small group of people doing it because it does take a while to become familiar with this disease and the workflows. So, I think it’d be hard if every primary doctor had one COVID patient, it’s hard for them to really stay on top of all the data. But …you could absolutely have a group of interested primary care doctors working CROWN clinics.” -Provider 5
**Support for mental health needs –** *Degree to which clinic supports mental health needs, including facilitating connection to behavioral health* Largely favorable perceptions across patients who reported emotional benefit from clinical interaction and providers who responded to loneliness during clinic encounters	“I... wanted to know… what state I was, if it was improving or not …and also, I was always alone in my room, they would talk to me, [laughs] felt like I wasn’t alone.” - Patient 13 “…they would call us… one would feel calmer…it did help me a lot. ...like the information they would give us and…one would do what one was told, ‘you can do this and this’…this helped us…to cope.” -Patient 21	**“**What I do find, when I see patients in CROWN, is a lot of emotional support. …a lot of these patients have been quarantined from other human beings for the past few weeks, and they’re really lonely. They’re just grateful for the opportunity to sit in front of another human and speak to them.” –Provider 4
**Predominant use of virtual encounters –** *Acceptance of video and telephone encounters as the primary modality of clinical care, with escalation to in-person care only as needed* Mixed acceptance across patients and providers, most of whom recognized need for virtual visits to reduce transmission risk, though some patients preferred in-person encounters to ensure accuracy of physical exam	“…the video call is better because I don’t have to go out or maybe contaminate another different person…[on] my way to the hospital…I don’t like to take that risk” – Patient 09 “[I prefer appointments] in person. See, by phone don’t make no sense to me. So, I go in person I can explain to the doctor it hurt me right here and that’s what happened…It’s a lot different talking in person over talking by phone.”- Patient 12	“I prefer videos when I can because there’s a lot to be gained when you can actually just eyeball a person…” -Provider 1
**Incorporation of pulse oximetry –** *Inclusion of home pulse oximetry for medium and high risk patients with data sharing as a part of a standard protocol* Favorable perceptions across patients and providers who reported it supported decision-making	“Even when I was feeling healthy, I was still using it just to make sure that it didn’t…go below whatever that number, 95, 96 or something… it was straight forward and they sent…an instruction that was very thorough.” -Patient 3	“If a patient tells me they’re feeling short of breath and their pulse ox is below 95, then I want them to come in to recheck the pulse ox with ours and take a listen to their lungs and that kind of thing.” -Provider 8
**Support for social needs** ***–*** *Degree to which CROWN was able to meet the social needs of its patients* Favorable perceptions in patients, some of whom reported using these services; mixed perceptions amongst providers, some of whom felt patient needs surpassed what they were able to offer	“Yes, they would always ask me if I needed help to do my grocery shopping, you know, when you need to buy things at the store, but you cannot leave the house, but I would always tell them that I had people helping me.” - Patient 6	“…some of the social challenges that some of our patients may have… [there are some] that are like, ‘No, I don’t have a doctor. I don’t have a clinic. I don’t have anybody to help me with getting food and water. And I’m living in a hotel.’…In the immediate moment, if I feel like somebody needs something that we cannot get for them at home and I’m worried about them, I direct them to the ER. And I write a note saying why. Whether it be for admission, for socioeconomic issues, or lack of social support or whatnot, or just elderly not going to do well at home, can’t get home health in to check on them.” –Provider 2
**Continuity of specialist care –** *Integration of specialty care into overall care during COVID-19* Unfavorable perceptions in a subset of patients with comorbid disease; mixed perceptions from providers who reported variation in their ability to facilitate specialty care.	“They referred me to my oncologist. My oncologist referred me back to the CROWN clinic, so basically I had people refer me back and forth to one another, but I never got good answers…so my answers are always well its new, well we don’t know much, and I just, I think that’s a cop out.” –Patient 11	“So sometimes, I’m communicating with the specialists to advocate for the patient to be able to come back into their care…I have to talk to the hospital epidemiologist and then loop in…the specialists, and advocate for our patients to be released from isolation at Stanford. And it’s crazy.” - Provider 7

Acceptability of other aspects was mixed. Patients overwhelmingly reported the frequency of outreach was appropriate, apart from one asymptomatic patient who felt outreach was too frequent. For providers, some debate existed as to the appropriate frequency of outreach, with a few providers questioning whether a proactive outreach approach was the optimal use of scarce resources, noting that many patients felt fine when they were contacted.

In addition, while most patients accepted virtual care via video or phone, noting community-wide shelter-in-place orders and their own need to isolate given their diagnosis, two participants expressed concerns that remote care could contribute to missed important clinical changes. Providers usually preferred video to phone so they could “eyeball” a patient to visually assess their clinical status (Provider 1). In addition, a few providers also regretted the system’s inability to provide home social services to patients in a time of significant need—an ED visit was sometimes the only solution when a patient could not adequately care for him- or herself.

Finally, acceptability was limited by the challenges associated with specialist care, particularly for patients with serious comorbid conditions (i.e., cancer, post-transplant). Patients wanted to know what their COVID-19 diagnosis meant for them in terms of their pre-existing condition; providers’ efforts to connect with these specialists on behalf of patients were sometimes fruitless. Particularly challenging for providers was convincing specialists that patients were no longer infectious after their 10-day quarantine and therefore qualified for in-person specialty care.

## Discussion

The specialized COVID-19 clinic and RPM program was launched early in the COVID-19 pandemic to fulfill multiple stakeholder needs—primarily to promote access to non-emergency services for patients suffering from COVID-19 and secure patient and staff safety systemwide [30]. This convergent mixed methods analysis suggests stakeholder beliefs that the clinic reduced acute care utilization (ED and admission events) was not supported by the quantitative analysis. Rather, observed hospitalizations were actually higher in the enrolled group than the unenrolled group. Further, no difference was observed between groups for ED events that did not result in an admission. Despite these negative quantitative findings, qualitative data suggests healthcare access increased for patients with confirmed or suspected COVID-19 who were otherwise restricted from non-emergency care. Finally, the clinic’s unmeasured benefit supporting the safe reopening of health services systemwide was felt to be substantial.

These findings seem to contradict the reduction in acute care utilization previously seen following COVID-19 RPM efforts in which patients were enrolled following an ED encounter or hospitalization [11, 12]. Our analysis differed from these studies in that it focused on patients who were enrolled following an outpatient diagnosis. This difference, as well as variation in how RPM was implemented may contribute to our observation that hospitalization was higher in the enrolled patient group [22].

Such increase in the utilization of health services has been previously seen with increased healthcare access [35, 36]. Increased exposure to the health system through the COVID-19 clinic may have increased patient comfort with the health care system overall, thereby lowering patients’ perceived barrier to seeking a higher level of care. Though clinician perspectives presented here suggest increased utilization was often an appropriate escalation of care, we did find patients presenting to the ED for asymptomatic repeat COVID-19 testing in both the enrolled and unenrolled groups. This suggests increased opportunity for patient education regardless of enrollment status, and possibly at the time of their initial diagnosis.

At the time of the analysis, outpatient care for COVID-19 was also primarily supportive. Simply increasing the monitoring for clinical deterioration (rather than combining this effort with treatment) was perhaps unlikely in retrospect to change the clinical course of the disease. Instead, clinicians had more opportunity to identify worsening disease and therefore escalate patients to a higher level of care. Future work in this area should account for COVID-19 outpatient treatments that have become available since this analysis [37].

Other possible reasons for increased utilization in the enrolled group include increased patient psychological need for health services at a time of great vulnerability [38], ED physician bias to admit a patient sent in by another provider, and residual selection bias towards “sicker” patients in an enrolled population (i.e. patients with underlying disease not already accounted for in the analysis are more likely to opt into a monitoring program) [39].

The present work represents an example of a collaboration between researchers, clinicians and health system administrators to evaluate an ongoing initiative in order to inform future improvements and the direction of limited resources [40]. Evaluating the value of such a clinic from a mixed-methods, multi-stakeholder perspective is particularly important in this setting where typical value-oriented data (i.e., cost and outcomes) is missing or overshadowed by unmeasured benefits —for example, benefits from the gradual reopening of a health system following an initial lockdown where no comparator exists.

The present work is also notable in its examination of an important new use case for RPM: monitoring of acute infectious disease. All stakeholders largely believed that RPM added value in this clinical context, and patients and providers described how it shifted their care-seeking behavior and clinical decision-making, respectively. This novel use of RPM touches on growing trends to explore and expand “hospital-at-home” models in which patients who would otherwise meet inpatient criteria received care in the comfort of their own home, with the support of remote clinical experts [41–43]. Understanding the bridge between hospital-at-home and traditional RPM will be an important area for future research. Further, careful consideration should be given to which types of patients may benefit most from RPM given limited resources. For example, targeting clinic resources towards patients who are higher risk for adverse outcomes (given the link between increased risk and escalation of care demonstrated in Table 3), and/or with a lower level of health literacy may be more cost-effective than a uniform approach and is an area for future work.

Evaluation limitations include the retrospective case-control design in which patients had the option to opt into the program; we were also unable to verify through the data that each patient was equally offered enrollment following standard protocols. Our analysis therefore relies on successful matching across several variables. The absence of any statistically significant difference in these variables and acute care events unrelated to COVID-19 between these groups provides some reassurance against selection bias. Individuals without access to at least a phone were unreachable and therefore not enrolled, though prior work suggests this is a small population [44]. Manual chart review captured all ED encounters and hospitalizations that occurred within any health system in the area that used the predominant electronic health record system (Epic Systems), though uncaptured encounters outside this network were possible. We further note that diverse clinical circumstances involving a novel disease limited our ability to fully define “appropriate” care. Our assumption therefore focused on two extremes—an admission following an ED visit suggests that ED visit was likely appropriate, whereas an asymptomatic patient presenting to the ED for a repeat COVID-19 test was likely better served in an alternative setting. We recognize possible exceptions to these generalizations, including our inability to draw conclusions about cases between those extremes (i.e. value-added ED visits [21]). For these reasons, conclusions should be interpreted with caution.

## Conclusion

Acute care utilization data and multi-stakeholder interviews suggest heightened outpatient observation through a specialized COVID-19 clinic and RPM program may have contributed to an increase in appropriate acute care utilization. A reduction in inappropriate care utilization was not seen, despite provider and administrator beliefs in this benefit. The clinic’s role securing safety systemwide, leading to a gradual reopening of health services systemwide was endorsed as a primary, if unmeasured, benefit. Additional evaluation is needed to understand the growing role of RPM in the novel support of acute infectious disease.

## Supplementary Information

**Additional file 1.**

## Data Availability

The datasets generated and/or analyzed during the current evaluation are not publicly available due to institutional data use agreements but are available from the corresponding author on reasonable request.

## Abbreviations

RPM: Remote Patient Monitoring
ED: Emergency Department
ER: Emergency Room
SHC: Stanford Health Care
CROWN: Care and Respiratory Observation of patients With Novel coronavirus
