# Supplementary material for: A 360 degree mixed-methods evaluation of a specialized COVID-19 outpatient clinic and remote patient monitoring program
Source: BMC Prim Care. 2022 Jun 13;23:151. doi: 10.1186/s12875-022-01734-7 (PMC9189794; doi:10.1186/s12875-022-01734-7)
Supplement: Supplementary file 1 — Additional file 1. [file 12875_2022_1734_MOESM1_ESM.docx]

**Additional file 1**

**Appendix A. Clinic-developed COVID-19 Risk Stratification & Outreach**

**Appendix A1. Clinic-developed COVID-19 Risk Stratification Tool**

| **Category** | **Domain** | **Specific Item** | **Risk Points** |
| --- | --- | --- | --- |
| 1 | Age | <65 years old | 0 |
|  |  | $\geq$65 years old | 1 |
| 2 | Pre-existing Conditions | Immunocompromised | 1 |
|  |  | Moderate to severe Asthma | 1 |
|  |  | Chronic lung disease | 1 |
|  |  | Cirrhosis | 1 |
|  |  | Diabetes | 1 |
|  |  | Severe obesity (BMI>40 kg/m^2^) | 1 |
|  |  | Cardiovascular disease (including Hypertension) | 1 |
|  |  | Chronic kidney disease | 1 |
|  |  | Pregnancy | 1 |
| 3 | Clinical Severity | Mild illness | 0 |
|  |  | - Appears well and stable |  |
|  |  | - Dyspnea that is mild, intermittent or resolving |  |
|  |  | - Fevers intermittent |  |
|  |  | - Tolerating PO intake with normal urine output |  |
|  |  | - Pulse <100 (while afebrile) |  |
|  |  | - Resting SpO2 >96% and ambulatory SpO2 >94% |  |
|  |  | NOT Mild illness | 2 |
| 4 | Clinical Course | - Course is stable | 0 |
|  |  | - Course is worsening | 1 |
| **TOTAL RISK SCORE** | | **Low Risk Score = 0**  **Moderate Risk Score = 1-2**  **High Risk Score > 2** | |
| COVID-19 patients with Moderate and High Risk Scores are offered pulse oximeter for home monitoring, and have longer follow-up in the Stanford CROWN Clinic | | | |

**Appendix A2. Tailored Follow-up for Outpatients with COVID-19 in Stanford CROWN Clinic Based on Initial Risk Score**

| **Day of illness** | **Low Risk Score** | **Moderate Risk Score** | **High Risk Score** | **Hospital Discharge**  **Follow-up** |
| --- | --- | --- | --- | --- |
| 1 | **Intake phone or video call only**  If patient has problems or questions related to COVID-19, they will first contact the CROWN clinic  Patient can be given return to work letter with date 10 days after diagnosis | Intake phone or video call by nurse or clinical provider | Intake phone or video call by nurse or clinical provider | Intake phone or video call by nurse or clinical provider |
| 2 | Discharged from CROWN Clinic | - | - | - |
| 3 | - | Phone call  (nurse) | Phone call  (nurse) | Video visit  (clinical provider) |
| 4 | - | - | - | - |
| 5 | - | - | Video visit  (clinical provider) | Phone call (RN) |
| 6 | - | Phone call  (nurse) | - | - |
| 7 | - | - | - | - |
| 8 | - | - | Video visit  (clinical provider) | Phone call (RN)  Discharge phone call to answer patient's questions and provide return to work notice.  If patient still has concerning symptoms we will continue to follow. |
| 9 | - | Video visit  (clinical provider) | - | - |
| 10 | - | - | Video visit  (clinical provider) | Discharged from CROWN Clinic |
| 11 | - | - | - | - |
| 12 | - | Phone call  (nurse)  Discharge phone call to answer patient's questions and provide return to work notice.  If patient still has concerning symptoms we will continue to follow. | Phone call  (nurse)  Discharge phone call to answer patient's questions and provide return to work notice.  If patient still has concerning symptoms we will continue to follow. | - |
| 13 | - | - | - | - |
| 14 | - | Discharged from CROWN Clinic | Discharged from CROWN Clinic | - |
| Clinical follow-up tailored based on patient clinical status. Whenever possible, language concordant medical assistants, nurses or clinical provider interact with patient. Clinical providers are either physicians or advanced practice providers (APPs). | | | | |

**Appendix B. Technical Process to Create the Enrolled and Unenrolled Matched Cohorts**

We extracted electronic health record data of all patients who received a positive test result from a Stanford testing facility between May 1, 2020 and September 30, 2020. In addition to test result and date, we obtained demographic information including patient age, gender, race, postal code, and insurance type. We also obtained dates of ED and hospitalizations during the same period including encounter diagnosis to assess whether events were COVID-19 related or not; events were defined as COVID-19 related if any diagnostic codes related to an encounter included COVID-19.

SHC served as a primary testing site within the Bay Area, therefore positive test results included patients both affiliated with SHC and those with no prior affiliation. We defined affiliation based on institutional affiliation of the assigned primary care provider (PCP); patients without an assigned primary care provider were defined as unaffiliated.

Manual chart reviews were conducted by physician researchers (SV, MA, LB, DH, LV, KC with regular audits by SV) to identify emergency room and hospitalizations in the exclusion and observation periods that were not captured in the extracted dataset but were viewable within the electronic health record (Epic, Wisconsin, USA). Chart reviews were also used to assess presence of comorbidities based on the COVID-19 risk stratification protocol; we assigned a binary code to indicate presence or absence of these comorbidities.

From initial data pull of COVID-19 positive tests, there were 719 enrolled patients (Appendix B, Figure 1). From this group, 61 patients were removed for being enrolled <= 7 days after recent hospitalization for COVID, 18 patients were removed because they lived more than 50 miles from Stanford, 43 patients were removed because they were enrolled after evaluation cut-off date 9/2/2020 (which would not allow for 28 days of observation post enrollment), 0 patients were removed for being under the age of 18 prior to 6/1/2020 (when evaluation began), and 3 patients were removed for being hospitalized outside of SHC 7 days prior to the enrollment date. These same exclusion criteria were applied to the unenrolled patient group with a positive COVID-19 test, with 267, 295, 629, 584, and 5 patients being removed in each category, respectively.

In addition to the above exclusion criteria, an additional step was taken to narrow the enrolled patient dataset. To minimize 1) chart review burden and 2) the likelihood of unobserved events that took place outside the electronic health record, which were both greater for unaffiliated patients (as opposed to SHC-affiliated patients), we capped the unaffiliated, enrolled sample population to a random selection of 100 individuals over the age of 50 who were matched following the procedures below; affiliated patient sample population was not capped. This removed 457 patients from the enrolled population, as the majority of positive tests were in unaffiliated patients. The higher age cut off >50 years old increased their likelihood of falling into a higher risk category (medium or high), therefore increasing our likelihood of observing an event (ED or hospitalization) within the follow up period. This increased our ability to detect a difference between the enrolled and unenrolled patients as well as validate the clinical tool used to triage patients (Table 3). The retained number was sufficient for analytical purposes.

The matched cohort was finalized using the following process. To minimize the number of chart reviews for potential unenrolled patients regarding exclusion criteria and comorbidities, a list of up to 10 closest matches for each enrolled patient was made based on an exact match of home distance from hospital (0-15, >15-30, >30-50 miles), health system affiliation (Stanford academic, Stanford non-academic, unaffiliated), insurance type (private, Medicare, other) and race/ethnicity (Hispanic, White, Asian, other). Once matched on those four variables, the closest matches were determined by the closest match in age calculated as the absolute difference in age. A second round of matching was completed after chart reviews where patients were matched based on presence of any of the comorbidities used for risk stratification, in addition to an exact match of home distance from hospital, health system affiliation, insurance type and race. The closest match was selected on the basis of exact matching on the previous variables and having the closest absolute difference in age to the enrolled patient. Unenrolled patients who were matched previously to an enrolled patient were not reused. The order of matching to the enrolled patients was done in descending order of age based on their age as of June 1, 2020 (i.e. the oldest patients were matched first).

As described in the Results, Table 1 suggests no significant difference between the two groups in terms of age, comorbidities.

**
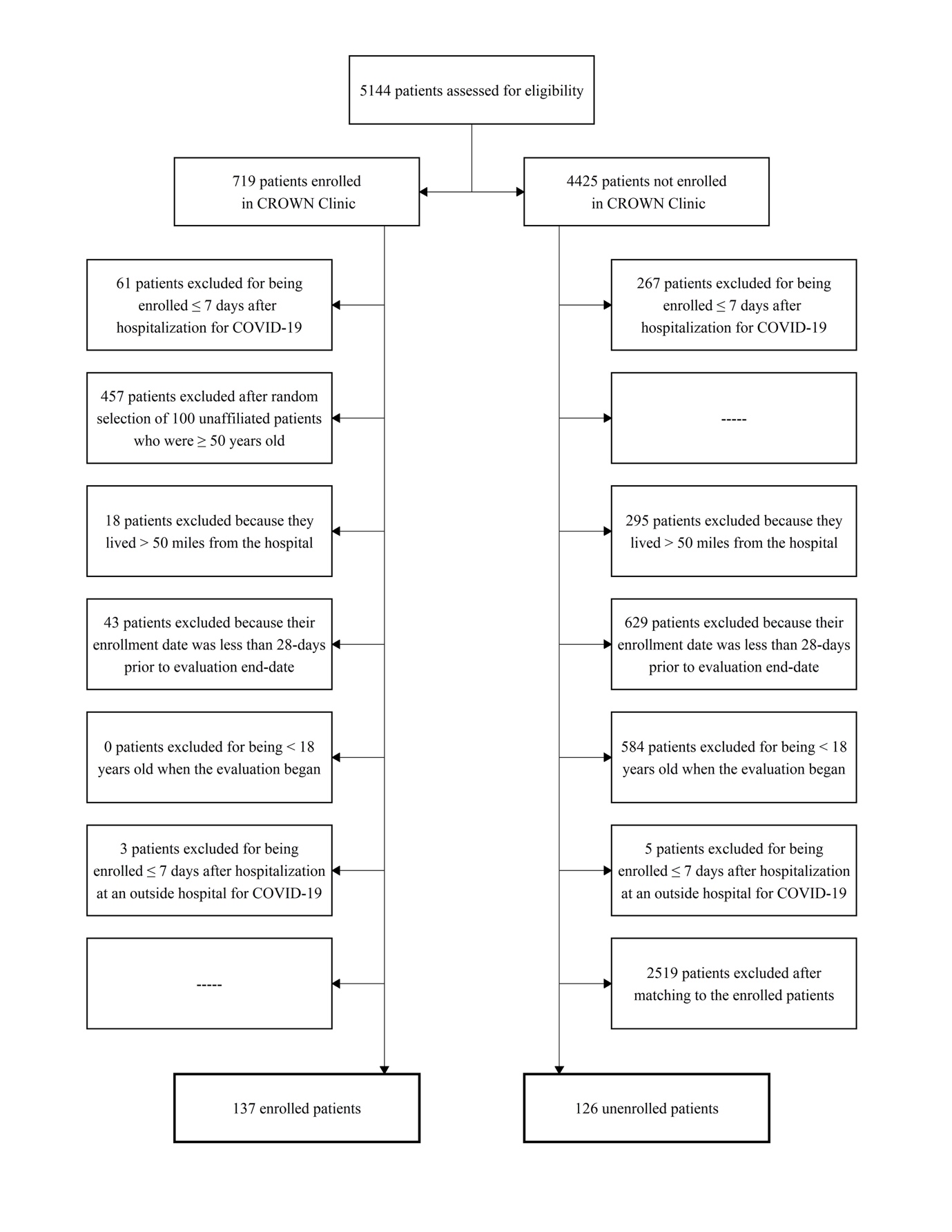
Appendix B, Figured 1. Exclusion Criteria to Create Final Enrolled and Unenrolled Groups**

**Appendix C. Interview Protocols Across Stakeholder Groups**

Patients were asked about their interaction with clinic providers, possible areas of improvement, use of a pulse oximeter, and about their care utilization outside the clinic; providers were asked about using remote monitoring to assess patient’s condition, clinical judgement in referring them to further care, care coordination, use of pulse oximeters, and risk stratification; finally, administrators were asked about the rationale behind the starting the clinic, the financial impact, and the sustainability of the model.

**Appendix C1. English Patient Qualitative Interview Protocol**

*Qualitative Interview Protocol for Patients treated for COVID-19 or their Caregivers*

| Information extracted from EPIC | |
| --- | --- |
| **MRN** |  |
| **Clinic name & institution**  **(Stanford/UHA)** |  |
| **CROWN clinic enrollment date** |  |
| **CROWN clinic discharge date** |  |
| **Sex** |  |
| **Age** |  |
| **Race** |  |
| **Ethnicity** |  |
| **Zip Code** |  |
| **Caregiver/patient interviewed** |  |

**Eligibility –** Patients or their caregivers discharged from CROWN and who were enrolled March 12 or later. Think about getting a diverse sample from low med high risk groups (at least 5 in each category as a start) as well as Spanish and non-Spanish speaking patients, diverse ages and genders.

**Voicemail**

Hi, I’m [introduce self], and I’m working with the Stanford CROWN clinic—the clinic that managed your care when you had COVID-19. I’m calling to see if you would be willing to have a short conversation with me about your experience in order to better improve the clinic’s services. When you’re available, could you please call me back at [phone number] ?

**INTRODUCTION**

Hi, my name is [NAME], and I’m calling from the Stanford School of Medicine. I’m working with the Stanford CROWN clinic—the clinic that managed your care when you had COVID-19. This clinic is new, so we want to find out how well it is working for patients. I am not part of your Stanford clinical care team, and your decision to chat with me will not have an impact on your care. Our goal is just to learn more about your experience with the care you received for COVID-19 and get your feedback, so we can share recommendations with the CROWN clinical team. These conversations usually take about 20 to 30 minutes; is this a good time for you to chat? (Okay to pause and ask for 5 minutes, if nearing end time.)

Before we start, we just wanted to let you know that anything you say will be kept **confidential**, which means that we will not share who you are when we share your feedback. We also like to audio record interviews to help us focus on you. Are you comfortable with me audio recording our discussion?

- Ok I’m turning on the recorder now.
- Can you say your name and that you agree to being recorded?

Thank you.

| **First, I’m going to ask you about your experience with the care you received at the Stanford CROWN clinic—the outpatient clinic that is just for COVID-19 patients.** | |
| --- | --- |
| 1. How did you first hear about the CROWN clinic? | (*If it comes up*) Tell me about your experience after you heard your COVID-19 test was positive.  How soon after your test did the CROWN clinic contact you?  Were you given instructions on what to do until you got the results of your test?  After hearing about the CROWN clinic, how did you expect them to help you? |
| 1. Tell me about your experience with the CROWN clinic. | What did they do for you?  Did you have in-person, video or phone visits?  Did you prefer telephone or video?  (*If they did not want video visits):* Is there a reason why you chose not to have video visits?  What worked well?  What did not work well?  Were there other services you wished you could have had at the CROWN clinic?  How did your experience with the CROWN clinic compare to your expectations for it? |
| 1. Tell me about your visits (calls) with providers. | How often did they call/speak to you? Did that feel like too much, not enough or just right?  Did you speak to the same provider each time?  Was there anything you would have liked to discuss that you did not? |
| 1. How helpful were the CROWN providers? | Was there anything they did that was particularly helpful?/ Did you learn anything new?  Was there anything you would have liked them to do differently?  Did you have questions for the CROWN providers?  *(If in Spanish*) Was there an interpreter present at those calls?  Was there anything you wanted to communicate to the CROWN clinic and weren’t able to due to language?  After discharge, did you still have questions about COVID-19 and your condition that you felt were still unanswered/ unclear? |
| 1. Did you receive an oximeter from the clinic? | *(If yes)* Can you tell me about how you used it?  Did you discuss your results with a CROWN clinic provider?  Was having the oximeter helpful for deciding whether or not to contact a provider? |
| 1. Did you ever have to get in touch with the clinic? | *(If yes)* For what reason? How did you do that? Did you have any trouble? |
| 1. Is there anything else about your experience with the CROWN clinic that you would like to tell me about? | Are there other ways your experience could have been improved? |

| **Now I’d like to ask you about any other care you received during this time aside from the CROWN Clinic.** | |
| --- | --- |
| 1. Do you have a primary care provider? | *(If no*) What provider do you usually go to or call if you want advice about a health problem or if you get sick?  Are you a Stanford patient? |
| 1. While you had COVID-19, did you see or contact your/a primary care doctor? | (*If no*) Why not?  (*If yes*) Did you contact them about your COVID-19 diagnosis or was it for something else?  During the time you were sick, would you have preferred to receive care from your primary care provider? Why/ why not?  How did your experience with the CROWN clinic compare to your other experiences receiving healthcare? |
| 1. Did you have any pre-existing conditions prior to testing positive for COVID-19? | (*If yes*) Do you have other providers that help you manage that condition?  Were you in contact with those providers after you tested positive for COVID-19?  Do you know if the CROWN providers were in contact with your (*specialist mentioned above*)? |
| 1. While you were sick, did you go to the emergency room or get admitted to the hospital? | *(If yes*) Which ED/ hospital did you go to?  Why did you decide to go?  How did you feel about having to go to the ED or hospital?  How did you feel about the care you received there versus the care you received from the CROWN providers? |
| 1. That’s all the questions I have, is there anything else you’d like to add? |  |

Thank you so much for your time!

**Appendix C2. Provider Qualitative Interview Protocol**

*Qualitative Interview Protocol for Clinicians working in the CROWN Clinic*

**Eligibility –** Clinicians (physicians, advanced practice providers, nurses, medical assistants) working at least 3 shifts within the CROWN Clinic since mid-May 2020 or involved in its ongoing operations. Think about getting a diverse sample including roles, age, experience, gender, race/ethnicity if available.

**INTERVIEW – Introduction**

First to **introduce ourselves**, we are exploring the impact of home monitoring for COVID-19 for the purpose of Quality Improvement. Our goal is to explore what you think about the CROWN Clinic COVID-19 home monitoring.

Before we start, we just wanted to let you know that our typical process is to keep everything you say **confidential**, which means that we make sure we are not sharing your identity when we share your insights. We also like to audio record interviews to help us focus on you. Are you comfortable with me audio recording our discussion?

Do you mind saying your full name and that you agree to being recorded?

| **Background information: I’d like to start with a little background information about you and your role in the CROWN clinic—the outpatient clinic that is just for COVID-19 patients.** | |
| --- | --- |
| First, could you tell me a bit about where you work outside the CROWN clinic and what you do day to day. | How do you split your time between usual work and the CROWN clinic?  In what ways is working at the CROWN clinic different to usual work? |
| In what ways do you think providing care to COVID-19 patients is working well with the CROWN clinic? | How well are video visits working?  How are you meeting the needs of non-English speakers? In what ways are their needs different? (social determinants of health?)  How well do you think the clinic is addressing patient concerns (emotional, financial, social etc)? |
| What do you see are the gaps in care for patients through the CROWN clinic? | Have you noticed any difference between patient groups? (**e.g. English, non-English speakers**, those with underlying health conditions, etc) Is it working better for some patients than others? Why?  Are there certain groups of patients who have declined the clinic’s services? Why?  How does the CROWN clinic provide care for patients who have serious **co-morbid illness**, such as patients with cancer or diabetes?  Is there anything you would have liked to do differently for these patients?  In what ways could protocols be improved? |
| Do you coordinate patient care with their primary providers? How/why not? | Are you able to do this for all patients or only Stanford patients?  Do you coordinate care with any other providers (e.g. cancer patients, co-morbidities)? How/why not? |
| **I now want to talk about some of the things that are specific or novel to the CROWN clinic, such as the pulse oximeters and proactive outreach to patients.** | |
| Tell me about how you have used the exertional pulse oximeters. | What challenges did you face in instructing patients to use pulse oximetry and report out results?  How has the use of pulse oximetry impacted your clinical decision-making? |
| How have you been able to follow the protocols for proactively reaching out to patients? |  |
| How well is the risk stratification working? |  |
| Is there anything else you think that is novel about the CROWN clinic? | Could this approach to monitoring patients with COVID-19 be done through usual primary care services? How/why not? |
| How do you as a provider feel about your ability to care for patients in the CROWN clinic model? | How has working in CROWN impacted you? (e.g. stress, safety, well-being) |
| Is there anything else you’d like to mention? |  |

Thank you so much for your time!

**Appendix C3. Administrator Qualitative Interview Protocol**

*Qualitative Interview Protocol for Administrators regarding CROWN*

**Eligibility –** Administrators with a decision-making or operational role related to the CROWN Clinic. Think about getting a diverse sample including roles, age, experience, gender, race/ethnicity if available.

**INTERVIEW – Introduction**

Thank you for speaking with me today. We are exploring the impact of home monitoring for COVID-19 for the purpose of Quality Improvement. Our goal is to explore what you think about the CROWN Clinic COVID-19 home monitoring.

Before we start, we just wanted to assure you that we will keep everything you say **confidential**, which means that we make sure we are not sharing your identity when we share your insights. We also like to audio record interviews to help us focus on you. Are you comfortable with me audio recording our discussion?

Do you mind saying your full name and that you agree to being recorded?

| **Background information: I’d like to start with a little background information about your role, including how it relates to the CROWN clinic.** | |
| --- | --- |
| First, could you tell me a bit about your role and the day to day activities you perform related to the CROWN clinic? | How have you engaged on the topic of the CROWN Clinic?  What role did you play in its inception in spring 2020?  What role do you play in its ongoing operations? |
| In thinking about the decision to launch the clinic, what considerations were most important?  *[Note to delay probes as long as possible; “We really want to hear from you.”]* | What role did **patient care** play in the decision to launch the clinic? For non-COVID-19 patients?  What role did **finances** play in the decision to launch the clinic?  What role did **provider/staff safety** play in the decision to launch the clinic? |
| Now that the CROWN clinic has been operational for six months, what do you think of this effort to carve out COVID-19 outpatient care from standard primary care? | What **benefits** did you see in carving out COVID-19 outpatient care from standard primary care?  What **drawbacks** did you see in carving out COVID-19 outpatient care from standard primary care?  What **changes** have taken place to improve CROWN operations?  Possible prompt areas: Patient care, finances, provider/staff safety, care coordination, equity |
| How has CROWN served the needs of vulnerable patients? | Non-English speakers?  What about patients with **co-morbid illness** (e.g. cancer)?  How does CROWN fit within Stanford Health Care’s efforts to **improve equity**? |
| What level of support exists to provide ongoing pulse oximeters to COVID-19 positive patients? | What are likely sources of future funding for these devices? |
| Do you see the CROWN model continuing at Stanford Health Care throughout the pandemic? | Why or why not?  What barriers exist to CROWN’s continuation?  What advice would you give other health systems who want to start a dedicated COVID-19 clinic? |
| Is there anything else you’d like to mention? |  |

Thank you so much for your time!

**Appendix D. Acute Care Events, Mortality and Duration of Admission Within a 28-Day Observation Period Not Related to COVID-19 by Enrolled and Unenrolled Patients**

There was no statistically significant difference in the proportion of patients who went to the ED or were hospitalized for non-COVID-19 related reasons between the enrolled and unenrolled patients (p=.32, p>.99, respectively), suggesting that these groups were reasonably balanced on more severe disease that might impact their COVID-19 disease severity or their general ED/hospital utilization. There was a significant difference between the number of non-COVID-19 ED-only events (p=.03).

| **Events During Observation Period** | **Enrolled** n = 137 (%) | **Unenrolled** n = 126 (%) | ***p*-value^a^** |
| --- | --- | --- | --- |
| **Patients with non-COVID-related ED-only^b^ events** | 12 (8.8) | 7 (5.6) | *p* = .32 |
| **Total non-COVID-related ED-only events** | 20 | 7 | *p* = .03 |
|  |  |  |  |
| **Patients with non-COVID-related hospital events** | 3 (2.2) | 2 (1.6) | *p* > .99 |
| **Total non-COVID-related hospital events** | 4 | 6 | *p* = .45 |
